# First person – Stephanie LaHaye and Uddalak Majumdar

**DOI:** 10.1242/dmm.040873

**Published:** 2019-06-24

## Abstract

First Person is a series of interviews with the first authors of a selection of papers published in Disease Models & Mechanisms, helping early-career researchers promote themselves alongside their papers. Stephanie LaHaye and Uddalak Majumdar are co-first authors on ‘Developmental origins for semilunar valve stenosis identified in mice harboring congenital heart disease-associated *GATA4* mutation’, published in DMM. Stephanie is a postdoc in the lab of Richard K. Wilson at the Institute for Genomic Medicine, Nationwide Children's Hospital, Columbus, USA, investigating the use of bioinformatic and genomic approaches to better understand how the coding and noncoding genome are associated with pediatric brain tumors. Uddalak is a postdoc in the lab of Vidu Garg at the Center for Cardiovascular Research, Nationwide Children's Hospital, Columbus, USA, investigating the mechanism of aortic valve calcification using genetic and proteomic approaches.


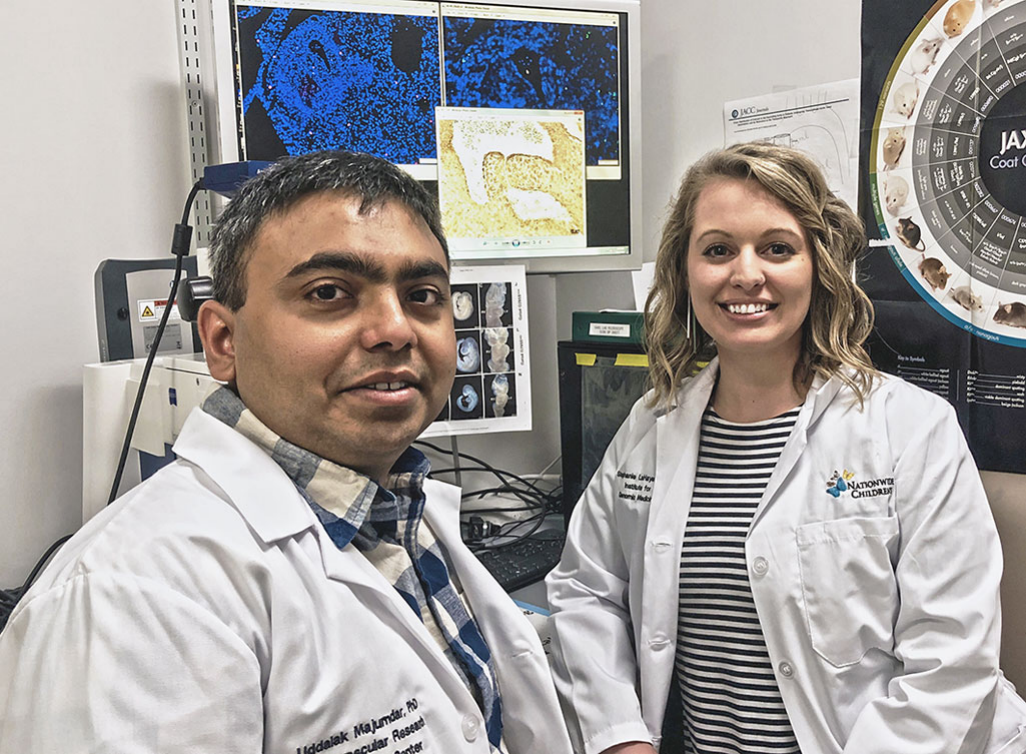


**Uddalak Majumdar and Stephanie LaHaye**

**How would you explain the main findings of your paper to non-scientific family and friends?**

SL+UM: In this study, we aimed to gain a clearer understanding of the underlying etiology of congenital semilunar valve stenosis through the study of a mutation in the *GATA4* gene, which was initially identified in a large family with inherited semilunar valve stenosis. The semilunar valves, which include the aortic and pulmonic valves, play an essential role in the unidirectional flow of blood from the ventricles to the outflow tract. Valvular stenosis occurs when the valve leaflets become thickened and stiff, which can leave them unable to properly open and close and so restricts blood flow from the heart. There are currently no pharmacological treatment options available to stop or reverse valve disease progression, which is why it is essential that we generate disease models to better understand disease mechanism and identify potential therapeutic targets. Using our novel mouse model containing the human disease-causing mutation, we were able to recapitulate the human valve disease phenotype and characterize the role of GATA4 in semilunar valve development and disease. Through the utilization of this model, we were able to identify key developmental deficits, including dysregulation of potentially targetable pathways that led to improper valve development and resulted in a stenotic valve. Ours is the first mouse model of congenital valve stenosis, to our knowledge, that was generated using a human disease-causing mutation, and we look forward to continuing to use this model to study valve disease.

**What are the potential implications of these results for your field of research?**

SL+UM: Through the use of our novel mouse model which contains an orthologous human disease-causing mutation, we were able to study the role of *GATA4* during semilunar valve development and disease. This novel mouse model is an excellent tool for researchers to study valve development and disease, and also provides support for the generation of future mouse models containing orthologous human mutations. In addition, we used this model to identify deficits in endothelial-to-mesenchymal transition (EMT), as well as abnormal proliferation and apoptosis. Previous studies have highlighted the role of *Gata4* in EMT during development of atrioventricular valves, and in this study we demonstrated that the role of *Gata4* in EMT is also important during development of semilunar/outflow tract valves. Our work also shows the *Gata4* mutation leads to a compensation from other cell types, due to the deficits in EMT; however incomplete compensation occurs, which could be one of the factors that leads to reduced penetrance. In addition, RNA-seq data highlighted the altered transcriptomic profile after disease onset in the *Gata4* mutant outflow tracts, which may help to identify potential therapeutic targets. This RNA-seq data is also available for other researchers to compare their datasets.

**What are the main advantages and drawbacks of the model system you have used as it relates to the disease you are investigating?**

SL+UM: The biggest advantage of our system is the ability to recapitulate a human valve disease phenotype in a mouse model through the use of an orthologous human disease-causing mutation. This is important because most human mutations are hypomorphic, i.e. they do not show complete loss of function. The use of a model harboring a point mutation allows us to identify the specific molecular deficits associated with the mutation, rather than a complete loss of an allele, which leads to a more thorough understanding of how mutations cause disease in humans. One disadvantage is that, although we have recapitulated a valve disease phenotype, in humans the *GATA4* mutation causes pulmonic valve stenosis, but we observed aortic valve stenosis in mice. The phenotypic differences that are observed could be attributed to the differences between humans and mice in hemodynamics, cardiac output and pressure that the valves experience during and after development. Although the affected valves are not same, this mouse is still a valuable model of congenital heart disease, as it is the first mouse model of a human mutation that causes congenital valve stenosis. In addition, as these valves are originated from the same cell population, and undergo similar signaling events during development, this model is a valuable tool to better understand the role of *GATA4* in semilunar valve development and disease.

**Figure:**
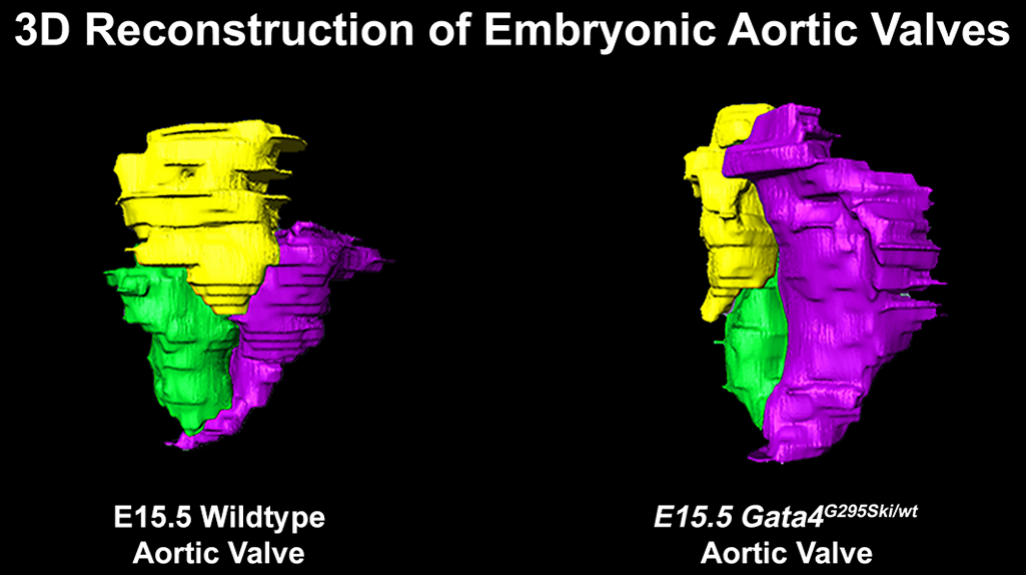
**3D reconstructed model of normal and diseased mouse embryonic aortic valves highlights the morphologic abnormalities associated with the *GATA4* mutation.**

“The biggest advantage of our system is the ability to recapitulate a human valve disease phenotype in a mouse model through the use of an orthologous human disease-causing mutation.”

**What has surprised you the most while conducting your research?**

SL: I was surprised by the volumetric differences that we identified at E13.5 in the *Gata4^G295Ski/wt^* aortic valve cushions compared to wild-type littermates through the use of 3D remodeling. Using histology alone it was not apparent that there were differences in the mutant cushions, but through 3D rebuilding of the cushions and volumetric quantification it was clear that there were differences. I was also surprised to see how dysmorphic the mutant E15.5 cushions were compared to controls, as this was also not apparent from the histological sections alone. I think that these findings highlight the importance of meticulous analysis of phenotypes and the incorporation techniques that are able to identify small changes using an unbiased approach. This will be an especially important consideration as more mouse models incorporating hypomorphic human mutations are generated with the onset of the CRISPR/Cas9 era.

UM: I was intrigued when we did not observe more significant differences in the cellular apoptosis and/or proliferation, but when we considered cellular density, it explained the reason behind the thickened valve volume. Also, the *in vivo* transcriptomic analyses at E15.5 revealed many differentially expressed genes between the wild-type and *Gata4* mutants, which led me wonder what the underlying molecular mechanisms of these gene regulatory networks are. Therefore, there are additional functional and expression-based assays that need to be performed to effectively model the differences associated with a disease-causing mutation in a cardiac transcription factor such as Gata4.

**Describe what you think is the most significant challenge impacting your research at this time and how will this be addressed over the next 10 years?**

SL+UM: The RNA-seq data demonstrated the altered transcriptomic profile after disease onset, but the identity of the cell type(s) in which this mutation is functioning improperly and leading to disease is not clear. Single-cell RNA-seq at E15.5, as well as at earlier developmental timepoints, will address some of these specific questions more clearly. Generation of a transgenic mouse line using CRISPR-Cas9 that harbors tagged knock-in and wild-type alleles followed by chromatin-immunoprecipitation sequencing will be instrumental to identify global changes in Gata4 targets during disease pathogenesis. This information will explain the disease mechanism more comprehensively and will guide us to develop novel targeted therapeutic strategies.

**What changes do you think could improve the professional lives of early-career scientists?**

SL: Early career investigators face numerous challenges as they transition into independent careers. One thing that has helped me as I navigate through my scientific career is the Research Institute Trainee Association (RITA) at Nationwide Children's Hospital (NCH). RITA is a group run by trainees that aims to support graduate students, postdocs and medical fellows within our research institute, and I currently serve as the 2019 RITA Co-Chair. RITA has provided numerous funding and training opportunities to me as I have transitioned from a graduate student to my postdoc position and has given me valuable insight into scientific career opportunities. RITA has also provided opportunities to engage in community outreach, teaching, networking and leadership roles. I highly encourage all young scientists to seek out trainee groups like this within their home institutions, as these groups can provide valuable support and excellent funding opportunities.

UM: As a postdoc and early-career researcher, I see that one can have multiple avenues to pursue their career. The most challenging in current times is to stay in academia and secure consistent extramural funding to support the lab and career. Therefore, more funding opportunities with directed mentoring plans will help a successful career transition. Being a postdoc in a well-funded lab and great institution like NCH has given me the perspective and opportunity to collaborate with multiple senior investigators within and outside NCH to perform high-throughput experiments. I highly recommend that graduate students and early- and mid-career trainees attend scientific conferences and actively participate in early-career events offered at the meetings, which will help progress both their professional and personal development.

“I highly recommend that graduate students and early- and mid-career trainees attend scientific conferences and actively participate in early-career events.” – *Uddalak Majumdar*

**What's next for you?**

SL: I have recently transitioned into the role of a postdoctoral scientist at the Institute for Genomic Medicine at NCH, where I work in a collaborative environment under the guidance of Drs Richard Wilson, Peter White and Elaine Mardis. I focus on the development and implementation of computational and bioinformatic approaches to better understand the role of the coding and noncoding genome in pediatric cancer. In my current position, I have the unique opportunity to be involved in a large institutional review board-approved multi-discipline translational research project, which includes collaborative efforts amongst oncologists, surgeons, pathologists, laboratory technologists, bioinformaticians, clinical directors and genetic counselors. This protocol enrolls patients with high-risk, relapsed/refractory or difficult-to-classify cancers, and aims to identify clinically meaningful results, including refining diagnosis, clarifying prognosis and/or providing a targeted treatment option. Although I have transitioned into research outside of the cardiovascular system, I am still implementing the skills and training that I gained during my doctoral work studying transcriptional regulation and molecular genetics, and I am excited to continue developing my computational skills and to apply them in a translational setting.

UM: I completed my Ph.D. in Biochemistry and Molecular Biology and then did a short postdoctoral training on vesicular protein trafficking in Dr Nava Segev's lab at the University of Illinois at Chicago, USA. I moved to Columbus and started my second postdoc in Dr Vidu Garg's lab at the Center for Cardiovascular Research in 2015. Currently, I am working on cardiac valve development and calcific aortic valve disease using multiple *in vitro* and *in vivo* models. My current focus is to identify novel signaling mechanisms, which are important for cardiac valve development and disease. Also, I plan to write career development grants, which will help my transition as an independent investigator.
